# Status Epilepticus Enhances Depotentiation after Fully Established LTP in an NMDAR-Dependent but GluN2B-Independent Manner

**DOI:** 10.1155/2016/6592038

**Published:** 2016-01-03

**Authors:** Xiati Guli, Tursonjan Tokay, Timo Kirschstein, Rüdiger Köhling

**Affiliations:** ^1^Oscar Langendorff Institute of Physiology, University of Rostock, Gertrudenstraße 9, 18057 Rostock, Germany; ^2^Center for Life Sciences, Nazarbayev University, 53 Kabanbay Batyr Avenue, Astana 010000, Kazakhstan

## Abstract

N-Methyl-D-aspartate (NMDA) receptor-dependent long-term potentiation (LTP) can be reversed by low-frequency stimulation (LFS) referred to as depotentiation (DP). We previously found GluN2B upregulated in CA1 neurons from post-status epilepticus (post-SE) tissue associated with an enhanced LTP. Here, we tested whether LFS-induced DP is also altered in pathological GluN2B upregulation. Although LTP was enhanced in post-SE tissue, LTP was significantly reversed in this tissue, but not in controls. We next tested the effect of the GluN2B subunit-specific blocker Ro 25-6981 (1 *μ*M) on LFS-DP. As expected, LFS had no effect on synaptic strength in the presence of the GluN2B blocker in control tissue. In marked contrast, LFS-DP was also attained in post-SE tissue indicating that GluN2B was obviously not involved in depotentiation. To test for NMDA receptor-dependence, we applied the NMDA receptor antagonist D-AP5 (50 *μ*M) prior to LFS and observed that DP was abolished in both control and post-SE tissue confirming NMDA receptor involvement. These results indicate that control Schaffer collateral-CA1 synapses cannot be depotentiated after fully established LTP, but LFS was able to reverse LTP significantly in post-SE tissue. However, while LFS-DP clearly required NMDA receptor activation, GluN2B-containing NMDA receptors were not involved in this form of depotentiation.

## 1. Introduction

Synaptic plasticity is the key mechanism of information storage in the brain [1]. While there is no doubt that activation of postsynaptic N-methyl-D-aspartate (NMDA) receptors is pivotal for induction of many forms of synaptic plasticity in the hippocampus, the subtype-specific role of these receptors with respect of direction of plasticity is highly debated. In the hippocampus as well as the cortex, GluN2A was initially found to be related to long-term potentiation (LTP) in contrast to GluN2B favoring long-term depression (LTD) [2–4], but this view has been questioned by subsequent studies [5–8]. Moreover, GluN2B overexpression or reduced degradation of GluN2B was in fact associated with enhanced CA1-LTP [9–11]. In addition, we have recently found an upregulation of GluN2B subunits in CA1 neurons from post-status epilepticus (post-SE) rats, leading to enhanced TBS-induced LTP at Schaffer collateral-CA1 synapses [12].

LTP can be reversed by neuronal activity [13, 14] referred to as depotentiation (DP), and common protocols for DP are typical LTD-inducing paradigms such as low-frequency stimulation (LFS). However, LFS appears to be effective only during a narrow time window since DP was not obtained when LFS was applied 30 min after LTP induction [15, 16]. Thus, the extent of LTP reversal is inversely related to the interval between LTP induction and DP [17, 18]. Notably, LFS appears to activate different pathways when delivered to potentiated synapses (i.e., mediating DP) or to naive synapses (i.e., leading to LTD). While AMPA receptor dephosphorylation and internalization are common aspects in both LTD and DP induction, there are significant differences in (1) the phosphatase mediating AMPA receptor dephosphorylation [19–22], (2) the GluA1 serine residue dephosphorylated [23, 24], and (3) the enzymatic cascade involved in AMPA receptor trafficking [25, 26].

As with LTD, there is also a debate whether or not DP might be attributed to activation of a specific GluN2 subunit. The evidence so far rather points to an involvement of GluN2A [27, 28]. However, DP has not been tested in tissue with GluN2B overexpression or pathological upregulation, and, on the other hand, LTD was not changed under these circumstances [11, 12]. Since LTP was enhanced in post-SE tissue with pathological GluN2B upregulation [12], we hypothesized that DP might by unaltered or—even more likely—reduced in synapses prone to LTP. Unexpectedly, we found DP to be significantly enhanced in post-SE tissue, and this enhancement required NMDA receptor activation but was preserved after pharmacological GluN2B inhibition.

## 2. Materials and Methods

### 2.1. Status Epilepticus* In Vivo*

The muscarinic agonist pilocarpine was used to induce status epilepticus (SE) in male Wistar rats (30–33 days; Charles River, Sulzfeld, Germany) as described previously [12, 29]. All procedures were performed according to national and international guidelines on the ethical use of animals (European Council Directive 86/609/EEC). All efforts were made to minimize animal suffering and to reduce the number of animals used. In order to reduce peripheral cholinergic effects, rats were first given methyl-scopolamine nitrate (1 mg/kg, i.p.) 30 min prior to pilocarpine treatment. Then, pilocarpine hydrochloride (340 mg/kg, i.p.) or saline (referred to as control animals) was applied, and the animals were carefully monitored to observe spontaneous seizures with progression into SE. The onset of SE was determined when an animal had a stage 4 or 5 seizure [30] that was followed by continuous epileptic motor activity without showing any reaction to sensory stimuli such as gently touching against the whiskers. When SE did not develop within 60 min, rats were given a second pilocarpine dose (170 mg/kg, i.p.). In order to terminate SE after 40 min, rats received a 500 *μ*L bolus injection of diazepam solution (Ratiopharm, Ulm, Germany, 5 mg/mL, i.p.). Occasionally, diazepam had to be reinjected in order to stop seizure activity. Finally, the rats were fed with 5% glucose solution for 1 day and kept in separate cages.

### 2.2. Slice Preparation and Maintenance

Hippocampal slices were prepared using 2–10-month-old male post-SE and control rats (i.e., 1–3 months after SE). After deep anesthesia with diethyl ether, rats were decapitated and the brain was rapidly removed and submerged into oxygenated ice-cold dissection solution containing 125 mM NaCl, 26 mM NaHCO_3_, 3 mM KCl, 1.25 mM NaH_2_PO_4_, 0.2 mM CaCl_2_, 5 mM MgCl_2_, and 13 mM D-glucose (95% O_2_, 5% CO_2_; pH 7.4; Osm 306–314 mosmol/kg). Horizontal brain slices (400 *μ*m) of the hippocampus were prepared using a vibratome (Campden Instruments, Loughborough, UK), and slices were then transferred into a holding chamber containing artificial cerebrospinal fluid (ACSF) containing 125 mM NaCl, 26 mM NaHCO_3_, 3 mM KCl, 1.25 mM NaH_2_PO_4_, 2.5 mM CaCl_2_, 1.3 mM MgCl_2_, and 13 mM D-glucose (Osm 306–314 mosmol/kg). Slices were continuously bubbled with 95% O_2_ and 5% CO_2_ to maintain the pH at 7.4 and were allowed to recover at room temperature (20–22°C) for at least 1 hour before being transferred into recording chamber.

### 2.3. Electrophysiological Recording and Induction of Synaptic Plasticity

Hippocampal slices were transferred into an interface chamber and continuously superfused with oxygenated ACSF at a flow rate of 2 mL/min with a volumetric infusion pump MCM-500 (MC Medicine Technique GmbH, Alzenau, Germany) and the solution temperature was controlled at 32 ± 1°C by (npi Electronic GmbH, Tamm, Germany). The experiments started after an equilibration time of at least 30 min. Field excitatory postsynaptic potentials (fEPSPs) were recorded using borosilicate glass pipettes (2-3 MΩ, pulled with PIP5 from HEKA Elektronik, Lambrecht, Germany) filled with ACSF. Stimulating and recording electrodes were placed into CA1 stratum radiatum. Bipolar stimulation was performed with platinum wire electrode and applied to Schaffer collaterals with ISO-STIM01M stimulus isolator (npi Electronic GmbH, Tamm, Germany). Paired-pulse stimulation (interstimulus interval 40 ms) triggered by the Master-8 stimulator (A.M.P.I., Jerusalem, Israel) was used in order to calculate the paired-pulse ratio (PPR). The Schaffer collateral pathway was stimulated at a rate of 0.033 Hz with the baseline stimulation strength adjusted to 30–40% of the maximal fEPSP amplitude.

For LTP induction, a theta-burst stimulation (TBS) protocol consisting of 10 trains with 5 stimuli at 100 Hz (200 ms apart) was used. After full establishment of LTP (i.e., after 60 min), a low-frequency stimulation (LFS) paradigm (1 Hz, 900 stimuli, 15 min) was delivered in order to reverse LTP. LFS-induced depotentiation (LFS-DP) was assessed as the fEPSP at 60 min following LFS (i.e., after 135 min of the total experiment). For these synaptic plasticity experiments, only 2–4-month-old animals were used. In addition to synaptic plasticity experiments, we also performed control experiments in order to confirm the effect of Ro 25-6981. To this end, slices from control and post-SE animals were incubated with CNQX (10 *μ*M) and gabazine (1 *μ*M) in Mg^2+^-free ACSF. Following stimulation of Schaffer collaterals, NMDA receptor-mediated fEPSPs (NMDA-fEPSPs) were obtained. Under these conditions, we observed a small but consistent increase of NMDA-fEPSPs (similar for control and post-SE tissue: 119 ± 6%, *n* = 6 in control and 119 ± 2%, *n* = 7 in post-SE within 40 min). We therefore performed interleaved time-control experiments for normalizing the data obtained with NMDA receptor antagonists. To study the sensitivity of NMDA-fEPSPs, we added first Ro 25-6981 (1 *μ*M), and after 15 min D-AP5 (50 *μ*M) was added to entirely block the NMDA-fEPSP. After D-AP5, however, we occasionally observed small residual components that were regarded as non-NMDA receptor-dependent potential and therefore subtracted from all precedent fEPSPs. Hence, data are presented as the percentage of the D-AP5-sensitive fEPSP. These control experiments confirming the effect of Ro 25-6981 were performed in 8–10-month-old animals.

Recording signals were amplified and filtered at 1 kHz by an EXT-10-2F (npi Electronic GmbH, Tamm, Germany). Analog data were digitized with a Micro1401 analog-to-digital converter (Cambridge Electronic Design, Cambridge, UK) and stored for offline analysis using Signal 2.16 software (Cambridge Electronic Design, Cambridge, UK). The specific NMDA receptor antagonist D-2-amino-5-phosphonopentanoate (D-AP5) and the GluN2B-specific blocker Ro 25-6981 [(*α*R,*β*S)-a-(4-hydroxyphenyl)-b-methyl-4-(phenylmethyl)-1-piperidinepropanol maleate] were purchased from Tocris (Bristol, UK). All other chemicals used for physiological solutions were purchased from Sigma-Aldrich (Taufkirchen, Germany).

### 2.4. Statistical Analysis

All data are expressed as mean values and the standard error of the mean. Statistical comparison was performed using Student's paired two-tailed *t*-test, ANOVA, or Mann-Whitney *U* test (as indicated) with the level of significance set to *P* < 0.05. Significant differences were indicated with asterisks in all figures (^*∗*^ *P* < 0.05, ^*∗∗*^ *P* < 0.01).

## 3. Results

### 3.1. Enhanced LFS-Induced DP in Post-SE Tissue

**Figure 1:**
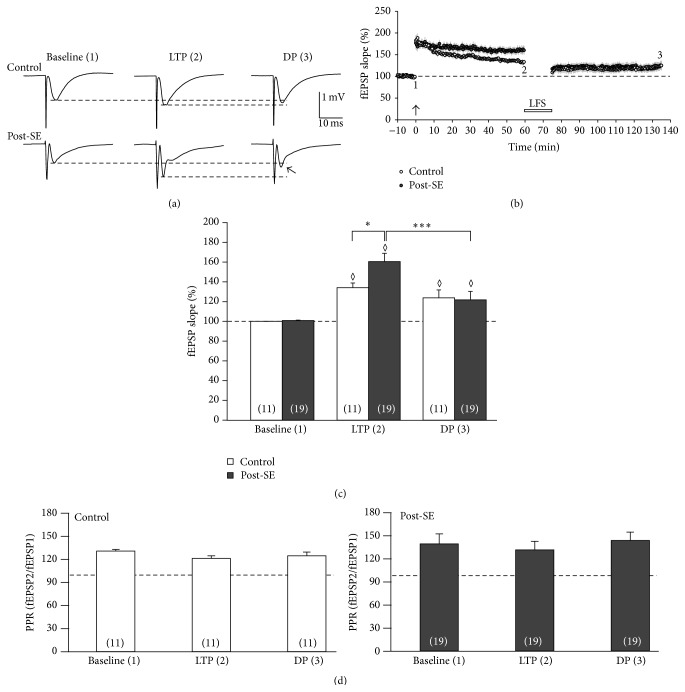
LFS-induced depotentiation (DP) in post-SE tissue. (a) Sample traces taken at baseline (timepoint 1 in panel (b)), directly before low-frequency stimulation (i.e., fully established LTP, timepoint 2 in panel (b)), and at the end of the experiment (i.e., depotentiation, DP, timepoint 3 in panel (b)). (b) Time course of the experiment showing the relative fEPSP slope (in % baseline). Following 10 min baseline, theta-burst stimulation (indicated by arrow) was applied to induced LTP which was allowed to develop for 60 min. Then, LFS was applied in order to depotentiate synapses again. The effect of LFS-induced DP was assessed after a follow-up of another 60 min (i.e., at 135 min after LTP induction). While there was a significant difference in LTP between control (open symbols) and post-SE tissue (closed symbols), LFS caused DP only in post-SE tissue, but not in controls. (c) Bar graph summarizing the relative fEPSP slopes (in % baseline) for three different timepoints (baseline, LTP, and DP). Diamonds indicate significant differences against baseline. Asterisks indicate significant differences as indicated by the brackets. (d) Paired-pulse ratio (PPR) of synaptic transmission following double-pulse stimulation (interstimulus interval 40 ms) for control (open bars) and post-SE tissue (closed bars) at three timepoints (baseline, LTP, and DP).

The aim of this study was to investigate low-frequency stimulation-induced depotentiation (LFS-DP) at Schaffer collateral-CA1 synapses in control and post-status epilepticus (post-SE) rats. Since GluN2B was upregulated in post-SE tissue leading to enhanced LTP at Schaffer collateral-CA1 synapses [12], we hypothesized that LFS-DP might by unaltered or even reduced at these synapses. To test this, we first induced robust long-term potentiation (LTP) using a theta-burst stimulation (TBS) paradigm in tissue from control and post-SE rats. As shown in Figure 1(b), TBS induced a long-lasting increase of the fEPSP slope in controls and even more so in post-SE tissue. After 60 min following TBS, we obtained significantly enhanced LTP levels in post-SE slices (closed symbols, 161 ± 8% of baseline, 60 min after TBS, *n* = 19) as compared to controls (open symbols, 134 ± 5% of baseline, *n* = 11, *P* < 0.05, Figure 1(c)) confirming our previous results [12]. Then, LFS was applied for 15 min, and fEPSPs were followed up again for another 60 min. At the end of this prolonged recording, we observed that LTP was significantly reversed only in post-SE tissue (122 ± 9% of baseline, *P* < 0.05 versus pre-LFS), but not in controls (124 ± 8% of baseline, *P* = 0.301 versus pre-LFS). In addition, the fEPSP slopes at the end of the experiment (i.e., 60 min after LFS) were still significantly larger than under baseline conditions (see diamonds in Figure 1(c)). Both TBS and LFS did not change the paired-pulse ratio (PPR) significantly, indicating the postsynaptic origin of the observed changes (Figure 1(d)). Hence, while LFS failed to depotentiate Schaffer collateral-CA1 synapses under control conditions, it did significantly reverse LTP in post-SE tissue.

### 3.2. NMDA Receptor Involvement in LFS-DP

**Figure 2:**
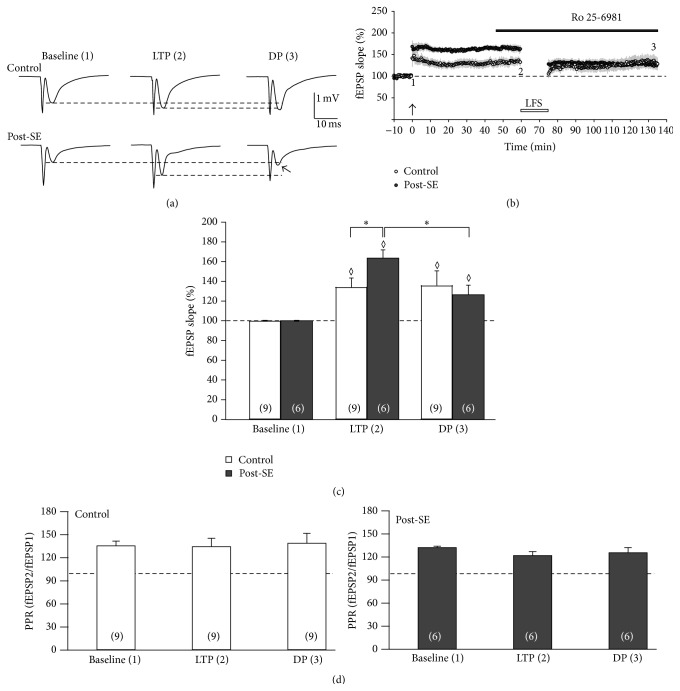
LFS-induced DP in epileptic tissue is not GluN2B-dependent. (a, b) Time course of the experiment showing the relative fEPSP slope (in % baseline). The GluN2B blocker Ro 25-6981 was applied as indicated by the bar. While LTP in post-SE tissue (closed symbols) was significantly enhanced as compared to control (open symbols) at timepoint 2 (60 min after TBS), there was no significant difference between both groups at timepoint 3 (i.e., DP) despite the presence of the GluN2B blocker. Sample traces (panel a) were taken at baseline (timepoint 1), directly before LFS (i.e., LTP, timepoint 2), and at the end of the experiment (i.e., DP, timepoint 3). (c) Bar graph summarizing the relative fEPSP slopes (in % baseline) for three different timepoints (baseline, LTP, and DP). Note that timepoints 2 and 3 are in the presence of the GluN2B blocker Ro 25-6981. Diamonds indicate significant differences against baseline. Asterisks indicate significant differences as indicated by the brackets. (d) Paired-pulse ratio (PPR) of synaptic transmission following double-pulse stimulation (interstimulus interval 40 ms) for control (open bars) and post-SE tissue (closed bars) at three timepoints (baseline, LTP, and DP).

In a previous report, we found that GluN2A was not altered in chronically epileptic tissue, but GluN2B was upregulated in these animals [12]. We therefore hypothesized that the difference in DP magnitude might be attributable to upregulated GluN2B subunits rather than to GluN2A which seems to be responsible for DP in control tissue [27, 28]. To test this, we repeated our experiments and applied the GluN2B subunit-specific blocker Ro 25-6981 (1 *μ*M) 15 min prior to LFS. As shown in Figure 2(b), TBS again led to a significantly higher LTP in post-SE (164 ± 8% of baseline, *n* = 6) as compared to controls (134 ± 9% of baseline, *n* = 9, *P* < 0.05, Figure 2(c)). However, as depicted in Figure 2(b), GluN2B inhibition by Ro 25-6981 did not block LFS-DP in post-SE tissue. On average, fEPSP slopes were significantly reduced by LFS to 126 ± 10% of baseline values (*n* = 6, *P* < 0.05 versus pre-LFS, Figure 2(c)) indicating that activation of GluN2B-containing NMDA receptors was not required for LFS-induced DP. In control tissue, LFS had no significant effect on the fEPSP slope (136 ± 15% of baseline, *n* = 9, *P* = 0.892 versus pre-LFS), consistent with a minor role of GluN2B-containing NMDA receptors in this tissue [12]. Similar to the results described above, the PPR was also stable during the course of the prolonged experiment indicating postsynaptically located expression of LFS-DP (Figure 2(d)).

**Figure 3:**
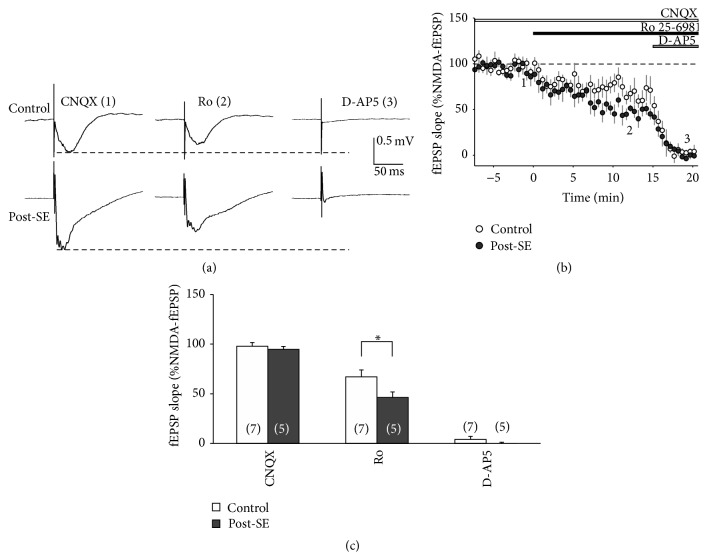
Ro 25-6981 efficiently reduce NMDA receptor-mediated fEPSPs. (a) Sample traces of NMDA receptor-mediated fEPSPs taken at baseline (CNQX, timepoint 1 in panel (b)), after application of Ro 25-6981 (1 *μ*M, timepoint 2 in panel (b)), and at the end of the experiment (i.e., after D-AP5, timepoint 3 in panel (b)). Note that, at baseline, slices were already incubated in Mg^2+^-free ACSF containing CNQX (10 *μ*M) and gabazine (1 *μ*M). (b) Time course of the experiment showing the relative fEPSP slope (in % baseline). Following baseline, Ro 25-6981 (1 *μ*M) was added to the bath and allowed to incubate for 15 min. Then, D-AP5 (50 *μ*M) was applied in order to block all NMDA receptor-mediated fEPSP. (c) Bar graph summarizing the relative fEPSP slopes (in % baseline) for three different timepoints (CNQX, Ro, and D-AP5). Asterisks indicate significant differences as indicated by the brackets.

Since 1 *μ*M Ro 25-6981 did not affect DP in either group, we were concerned about the efficiency of this compound under our conditions. Therefore, we performed control experiments with isolated NMDA receptor-mediated fEPSPs and tested the sensitivity of Ro 25-6981 (1 *μ*M). As is shown in Figure 3(b), NMDA receptor-mediated fEPSPs were sensitive to Ro 25-6981 and entirely blocked by D-AP5. Moreover, the residual NMDA-fEPSP following Ro 25-6981 was significantly larger in control compared to post-SE tissue (67 ± 7%, *n* = 7 versus 46 ± 6%, *n* = 5; *P* < 0.05, 2-way-ANOVA with Tukey post hoc test, Figure 3(c)). These data are consistent with enhanced GluN2B-related function [12] and confirm that 1 *μ*M Ro 25-6981 was efficient in the present study.

**Figure 4:**
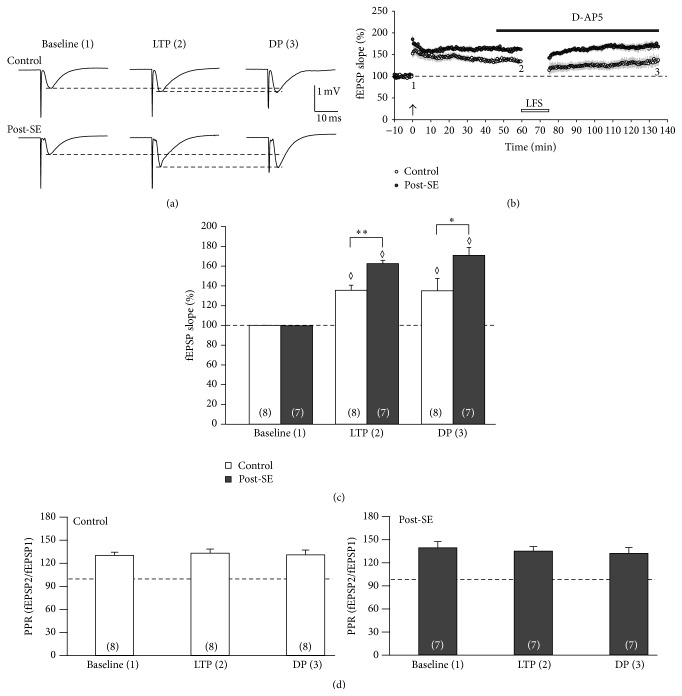
LFS-induced DP in epileptic tissue is NMDA receptor-dependent. (a, b) Time course of the experiment showing the relative fEPSP slope (in % baseline). The NMDA receptor blocker D-AP5 was applied as indicated by the bar. Now, the fEPSP slope in post-SE tissue (closed symbols) was significantly larger than in control tissue (open symbols) at timepoint 2 (60 min after TBS) but remained significantly enhanced at timepoint 3 (i.e., DP). Thus, D-AP5 blocked LFS-induced DP in post-SE tissue. Sample traces (panel a) were taken at baseline (timepoint 1), directly before LFS (i.e., LTP, timepoint 2), and at the end of the experiment (i.e., DP, timepoint 3). (c) Bar graph summarizing the relative fEPSP slopes (in % baseline) for three different timepoints (baseline, LTP, and DP). Note that timepoints 2 and 3 are in the presence of the NMDA receptor blocker D-AP5. Diamonds indicate significant differences against baseline. Asterisks indicate significant differences as indicated by the brackets. (d) Paired-pulse ratio (PPR) of synaptic transmission following double-pulse stimulation (interstimulus interval 40 ms) for control (open bars) and post-SE tissue (closed bars) at three timepoints (baseline, LTP, and DP).

Having found that GluN2B was not involved in LFS-DP, we wondered whether NMDA receptors are generally required for depotentiation. To address this question, we carried out a further set of experiments with the same protocol but replaced Ro 25-6981 by the nonspecific NMDA receptor blocker D-AP5 (50 *μ*M, Figure 4(b)). Hence, we added D-AP5 to the bath solution 15 min prior to LFS. Again, LTP was significantly enhanced in chronically epileptic tissue (post-SE: 163 ± 3%, *n* = 7; control: 136 ± 6%, *n* = 9; *P* < 0.01, Figure 4(c)), before LFS was applied. LFS, in turn, had no significant long-term effect on the fEPSP slope (post-SE: 171 ± 8%, *n* = 7, *P* = 0.399 versus pre-LFS; control: 132 ± 10%, *n* = 9, *P* = 0.774 versus pre-LFS). These results clearly confirmed the NMDA receptor-dependent nature of LFS-induced DP.  Figure 4(d) demonstrates stable PPR values following TBS and LFS, which is similar to all experiments above.

**Figure 5:**
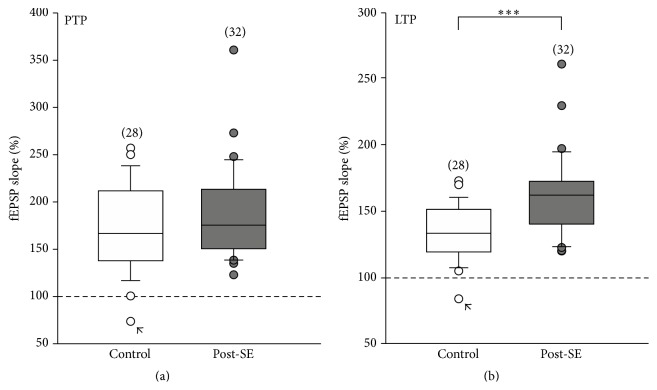
Comparison of posttetanic potentiation (PTP) and LTP in all experiments. (a, b) Box-whisker plots show posttetanic potentiation (PTP, panel (a)) and long-term potentiation (LTP, panel (b)) of all experiments presented in Figures 1, 2, and 4. Note the higher skewness of the data obtained from post-SE slices in both calculations. There was one control slice (indicated by an arrow) which showed a posttetanic depression and consequently LTD rather than PTP and LTP. While PTP was not statistically different between control and post-SE tissues, enhanced LTP in post-SE slices reached statistical significance (*P* < 0.001; Mann-Whitney *U* test).

When we compared the three experimental paradigms (i.e.m native conditions, Ro 25-6981, and D-AP5), we were concerned about the observed variance in posttetanic potentiation (PTP). On average, the PTP values were 173 ± 8% in control slices (*n* = 28) and 185 ± 9% in post-SE tissue (*n* = 32). This difference in PTP, however, was not statistically different. Next, we plotted box-whisker graphs for PTP and LTP, respectively, and found that the distribution of data obtained from post-SE tissue showed higher skewness and more extreme values (Figure 5). On the other hand, we could confirm that TBS-induced LTP was significantly enhanced in post-SE tissue (collectively 162 ± 5%, *n* = 32, as opposed to 134 ± 4%, *n* = 28, in all control slices, *P* < 0.001, Figure 5).

In conclusion, both TBS-induced LTP and LFS-induced depotentiation are enhanced in post-SE tissue from chronically epileptic rats.

## 4. Discussion

The aim of this study was to explore whether synapses prone to LTP by GluN2B upregulation can be depotentiated by low-frequency stimulation (LFS). We hypothesized that GluN2B upregulation in post-SE tissue causing enhanced LTP [12] would lead to impaired depotentiation (DP). However, we unexpectedly found DP to be significantly enhanced in this tissue. More precisely, DP was only accomplished in post-SE tissue, but not in controls. In addition, we found that DP in post-SE tissue did require NMDA receptor activation but was left intact after pharmacological GluN2B inhibition.

The major finding of our study was that—under control conditions—LFS failed to depotentiate synapses in a state of fully established LTP, while DP could be induced in post-SE tissue. The lack of LFS-induced DP in control synapses is consistent with previous reports. Thus, it has been shown that LTP reversal appeared only during a narrow time range after LTP induction and the extent of depotentiation was inversely related to the interval between LTP induction and LFS [15, 16, 31]. However, this may in part be an issue of the DP protocol, because fully established LTP (i.e., 60 min after induction) was demonstrated to be reversed by high-intensity paired-pulse LFS [28]. The same study demonstrated that DP was inhibited by low-molecular Zn^2+^ (30 nM), a voltage-independent GluN2A antagonist, pointing to a GluN2A-dependent mediation, while the GluN2B antagonist Ro 25-6981 had no effect on LTP reversal [28]. In another report, the preferential role of GluN2A in DP was further supported by experiments using NMDA application referred to as chemical DP [27], but it is also known that DP is an age-dependent synaptic property [16]. Taking this argument, GluN2B abundance showing a natural decline during development appears to correlate with the propensity of synapses to express DP. In this context, it is important to note that our experiments were performed during the chronic stage of pilocarpine-induced epilepsy, that is, in 2–4-month-old animals. Consistent with the idea of age-related decline of DP, the phenotypic regression of epileptic tissue to a developmentally immature stage with predominantly GluN2B expression (for review, see [32]) was in fact associated with an enhanced LFS-induced DP. Thus, our data suggest that adult tissue with—in this case pathological—GluN2B upregulation expressed DP to a similar degree to immature control tissue. While DP was definitely NMDAR-dependent, the direct requirement of GluN2B receptors was not supported by our experiments. Rather, it appears that downstream signaling mechanisms may be altered concomitantly with NMDA receptor subunits. For instance, a recent study indicated that muscarinic acetylcholine receptor (mAChR) activation interfered with the association between GluN2B and the Ras-specific guanine nucleotide-releasing factor 1 (RasGRF1) and treatment with the mAChR antagonist scopolamine increased the involvement of GluN2B in LTD induction [6]. While these experiments were unable to discern the mAChR subtype involved, the M1-mAChR at least was downregulated in post-SE tissue [33]. Thus, it is conceivable that downregulation of mAChR in post-SE tissue could contribute to the resurgence of DP in these synapses. Alternatively, there is some evidence that DP induction involves adenosine A_1_ receptor activation [34, 35]. Although it is difficult to measure adenosine in post-SE tissue, there is one report showing an increased immunoreactivity for ecto-5-nucleotidase, the adenosine producing enzyme, in the chronically epileptic hippocampus [36] leaving the intriguing possibility that enhanced DP in post-SE tissue could be partly due to increased adenosine levels. Thus, the enhanced DP in chronically epileptic tissue could be interpreted as a homeostatic process, but the underlying mechanisms are downstream of the activation of presumably GluN2A-containing NMDA receptors.

One potential limitation of the present study is that the involvement of GluN2B has been determined on the basis of pharmacological experiments. During the last decade, a number of pharmacological studies have explored a differential role for GluN2A and GluN2B in LTP and LTD, respectively, but obtained somewhat contrary results [2–4, 6, 7]. This is partly explained by insufficient selectivity of GluN2A antagonists and the poor efficacy of GluN2B antagonists at triheteromeric NMDA receptors [37]. However, 1 *μ*M Ro 25-6981 is commonly used to block GluN2B-containing NMDA receptors, and functional overexpression of Ro 25-6981-sensitive NMDA receptor-mediated currents has been found at multiple synapses following status epilepticus [12, 38]. To deal with this pharmacological problem, genetic models have also been used to study the role of GluN2 subunits in LTP and LTD. While transgenic overexpression of GluN2B or impaired GluN2B degradation enhanced hippocampal LTP, LTD was not changed under these circumstances [9–11]. Importantly, this is also true in the case of pathological GluN2B upregulation seen in post-SE tissue [12], thus questioning the requirement of GluN2B in LTD induction. In addition, CaM kinase II inhibition decreased surface GluN2B and reduced LTP, but LTD was intact [8]. On the other hand, constitutively GluN2B-deficient mice do not survive into adulthood but showed no LTD as neonates [39], and conditional CA1-specific GluN2B knockout mice had impaired LTD [40]. Interestingly, a recent report—using again the pharmacological approach—focused on extrasynaptic GluN2B receptors and suggested that GluN2A activation was required for LTD, but extrasynaptic GluN2B determined the magnitude of LTD [41]. In summary, the direction and magnitude of synaptic plasticity seem to depend on the relative composition and postsynaptic localization of NMDA receptors, rather than on the mere presence or absence of an individual NMDAR subtype. Within this context, it is important to note that similarly altered NMDAR subtype expression levels were detected in both the pilocarpine epilepsy model [12, 29, 42] and specimens from human temporal lobe epilepsy patients (e.g., [43]). Since GluN2B upregulation in post-SE animals can be viewed as an acquired channelopathy, this tissue offers the opportunity to study NMDAR function without the need for subtype-selective blockers. Albeit our data were obtained with pharmacological tools with limitations discussed above, we have previously found that the sum of both the GluN2B-blocker-sensitive EPSP component and the GluN2A-blocker-sensitive EPSP component was equal to the D-AP5-sensitive EPSP suggesting that both EPSP components were quite disjunctive [12]. Hence, while this does certainly not exclude a low efficiency at heteromeric channels, it indicates that the error made with pharmacological tools was at least not substantial.

Unraveling the disturbed downstream mechanisms of NMDAR activation under pathological conditions will help understand both cognitive deficits in epilepsy and epileptogenesis. When comparing depotentiation and LTD, it is well known that both forms of dampening synaptic strength involve different signaling mechanisms. A number of differences have been identified so far, such as the serine residue of GluA1 receptors dephosphorylated after LFS [23, 24] and the signaling cascade involved in AMPA receptor trafficking [26, 27]. In addition, many enzymes involved in LTD do not appear to play a major role in DP like protein phosphatase 2A [20] and phosphoinositide 3-kinase *γ* [21] as well as the Janus kinase/signal transducer and activator of transcription [22]. In contrast, calcineurin A*α* mutant mice had normal LTD but impaired DP [19]. In conclusion, our study adds a further piece of evidence to this literature that LTD and DP are distinct synaptic processes since pathological GluN2B overexpression in post-SE tissue promotes DP but leaves LTD unaltered. The pathophysiological consequences of such enhanced DP in post-SE tissue, however, are less clear. Obviously, synapses are less capable of residing in a potentiated state, but whether or not this may perturb memory performance in live animals may not be answered by our study. Nonetheless, we believe that the enhanced DP contributes to an altered homeostasis of synaptic maintenance under pathological conditions potentially giving rise to unstable memory formation and retrieval.
